# Effect of shelf-storage temperature on degree of conversion and microhardness of composite restorative materials

**DOI:** 10.1186/s12903-023-02770-0

**Published:** 2023-01-31

**Authors:** Omar Abd El-Maksoud, Hamdi Hosni Hamdan Hamama, Ramy Ahmed Wafaie, Noha El-Wassefy, Salah Hasab Mahmoud

**Affiliations:** 1 Conservative Dentistry Department, Faculty of Oral and Dental Medicine, Delta University for Science and Technology, Gamasa, Egypt; 2 Conservative Dentistry Department, Faculty of Dentistry, Mansoura University, Mansoura, Egypt; 3 Dental Biomaterials Department, Faculty of Dentistry, Mansoura University, Mansoura, Egypt

**Keywords:** Storage temperature, Degree of conversion, Microhardness, Ormocer, Nanoceramic composite

## Abstract

**Background:**

The pre-cure temperature is considered an important parameter that affects the polymerization kinetics and the properties of composite restoration. As dissension exists about the effect of storing composite restorative materials in refrigerator, this study aimed to assess the effect of shelf-storage temperature on degree of conversion (DC) and microhardness of three composite restorative materials with different matrix systems.

**Methods:**

Three commercially-available composite restorative materials were used in this study; an Ormocer-based composite (Admira Fusion, Voco GmbH), a nanoceramic composite, (Ceram.X SphereTEC One, Dentsply Sirona GmbH), and a nanohybrid composite (Tetric N-Ceram, Ivoclar Vivadent AG). Regarding DC and microhardness tests, 60 disc-shaped composite specimens for each test were randomly divided into 3 groups (n = 20) according to the restorative material used. Each group was divided into 2 subgroups (n = 10) according to the composite storage temperature; stored at room temperature or stored in the refrigerator at 4°–5 °C. DC was evaluated using a Fourier-transform infrared spectrometer coupled to an attenuated total reflectance accessory. Microhardness was evaluated using micro-Vickers hardness tester under a load of 50 g with a dwell time of 10 s. The results were analyzed by ANOVA, post-hoc LSD, and independent t-tests at a significance level of *p* < 0.05.

**Results:**

Regarding DC test all groups showed statistically significant differences at both storage temperature. The Ormocer-based composite had the highest mean values. There was a statistically significant difference between all room-stored groups and their corresponding groups stored at refrigerator (*p* < 0.05). For microhardness test, all groups exhibited also statistically significant differences at both storage temperatures with the Ormocer-based composite having the highest mean values. A statistically significant difference between both room-stored and refrigerator-stored groups has been observed also (*p* < 0.05).

**Conclusions:**

Refrigeration of resin-composite might have a deleterious effect on DC and microhardness of the tested composite restorative materials with different matrix systems. Moreover, the differences in the formulations of composite matrix have a potential impact on DC and microhardness.

## Background

Dental amalgam was considered the most commonly used restorative material owing to its ease of use, high durability, and affordable cost [1]. However, dental resin composite has been widely used nowadays as a substitute for dental amalgam due to the concerns about the possible hazardous risks on human health through mercury content in the amalgam alloy as well as the unesthetic appearance [2]. Resin composite restorative materials have gained high acceptance and preponderance as they exhibited higher physical, mechanical, optical, thermal, and esthetic characteristics in addition to non-toxic and antibacterial properties [3–5].

Dental resin composite consists of a continuous phase of an organic resin matrix and dispersed phase of inorganic fillers [6]. The resin matrix constitutes of monomers, diluents, photoinitiators, accelerators, and coupling agents [7]. In the 1960s, polymethyl methacrylate (PMMA) was introduced to the dental market with certain limitations such as high shrinkage rate and improper viscosity. Consequently, bisphenol-A glycidyl methacrylate (Bis-GMA) monomer has been used to overcome these drawbacks [8, 9]. Triethylene glycol dimethacrylate (TEGDMA) and 2-hydroxyethyl methacrylate (HEMA) were utilized as diluents to decrease the viscosity of Bis-GMA for adequate clinical use [3]. A variety of ceramics, glasses, and silica particles with different sizes and morphologies were employed as fillers for resin composites [10].

Variable amounts of resin matrices and inorganic fillers are combined to fabricate dental composite formulations [4]. The organic matrix and the inorganic fillers are both linked together by silane coupling agent which enhances the particle wetting and maximizes the filler loading [11]. This organic-nonorganic coupling agent is bonded to the filler particles by its nonorganic end and bonds with the resin matrix by its organic end [12]. A strong bond between the polymer matrix and the filler particles is essential to assure a high level of performance from the composite restoration [13].

The most substantial developments in resin composite restorative materials were related to the fillers with less attention to improvements in the resinous matrix [14, 15]. Polymerization shrinkage and its associated stress was considered one of the serious drawbacks of resin composites related to the organic matrix causing loss of marginal integrity, gap formation, micro-leakage, recurrent caries, post restorative sensitivity, and pulpal irritation [16, 17]. The recent enhancements are focused mainly on the polymeric matrix to provide systems with less polymerization shrinkage. Several low-shrink composites were introduced to the dental markets; Ormocer (organically modified ceramic) is one of these materials which is considered as an alternative for Bis-GMA-based composites [18, 19]. This material can be properly described as a three-dimensionally cross-linked copolymer that combines organic and inorganic components at a nanoscopic scale through solution and gelation process. The Siloxane oligomeric nanostructure (Si–O–Si) is produced through the hydrolysis and the polycondensation of functionalized alkoxysilanes groups [20]. Initially, Ormocers were incorporated with conventional methacrylates, but recently, a material formulated with a completely Ormocer-based resin matrix has been developed [21].

Adequate polymerization is a fundamental factor in achieving optimal mechanical properties and enhanced clinical performance of resin composite materials [22]. DC affords a qualitative and quantitative indicator for the extent of the polymerization [23]. DC has a great influence on the resin composites characteristics such as flexural strength, dimensional stability, solubility, and the extent of discoloration and degradation [24]. Surface hardness also is an important property that enables the resin composite to resist plastic deformation, penetration and scratching which is necessary for esthetic properties and indicates the easiness of finishing and polishing procedures. The microhardness measurement of resin composite can be also a valuable indicator of DC and thus, the clinical success of resin composite restorations [25, 26].

The temperature of composite restorative materials before curing greatly influences the polymerization process, which in turn affects the properties of the resultant polymer [27]. A temperature between 4 and 20 °C is usually recommended for adequate composite storage to ensure maximum effectiveness [28]. Some clinicians store resin composites in the refrigerators at 2–5 °C with the objective of extending their shelf life particularly in hot climate regions [29]. Moreover, some manufacturers usually recommend keeping the composite syringes inside the refrigerator. However, a debate exists about the effect of storing composite restorative materials in the refrigerator. Therefore, this in-vitro study was conducted to assess the effect of storage temperature on DC and microhardness of three composite restorative materials with different matrix systems.

## Methods

### Restorative materials

In this current study, 3 composite restorative materials were investigated as follows; An Ormocer-basedcomposite (Admira Fusion, Voco GmbH, Cuxhaven, Germany), a nanoceramic composite (Ceram.X SphereTEC One, Dentsply Sirona GmbH, Konstanz, Germany), and a nanohybrid composite **(**Tetric N-Ceram. Ivoclar Vivadent AG, Schaan, Liechtenstein). Each restorative material was used according to manufacturers’ instructions. The full description of the materials used is presented in Table 1.

**Table 1:** Restorative materials used in the study

Restorative materials	Specification	Manufacturer	Composition			Batch no
			Matrix	Filler	Filler degr**ee**	
Admira Fusion	Nanohybrid Ormocer-based composite	Voco GmbH	Ormocer	Silicon oxide nanofiller, glass–ceramic filler	84% by wt	1939483
Ceram.X SphereTEC One	Nanoceramic composite	Dentsply Sirona GmbH	Methacrylate-modified polysiloxane, Poly-urethane methacrylate, Bis-EMA, TEGDMA	Prepolymerized spherical fillers, Barium-aluminum borosilicate glass, ytterbium fluoride, methacrylate functionalized silicon dioxide nanofiller	77–79% by wt	1908000044
Tetric N Ceram	Nanohybrid composite	Ivoclar Vivadent AG	UDMA, Bis-GMA, Ethoxylated Bis-EMA, TEGDMA	Barium glass, ytterbium trifluoride, mixed oxide, silicon dioxide prepolymers	80–81% by wt	X49739

### Study design

**Fig. 1:**
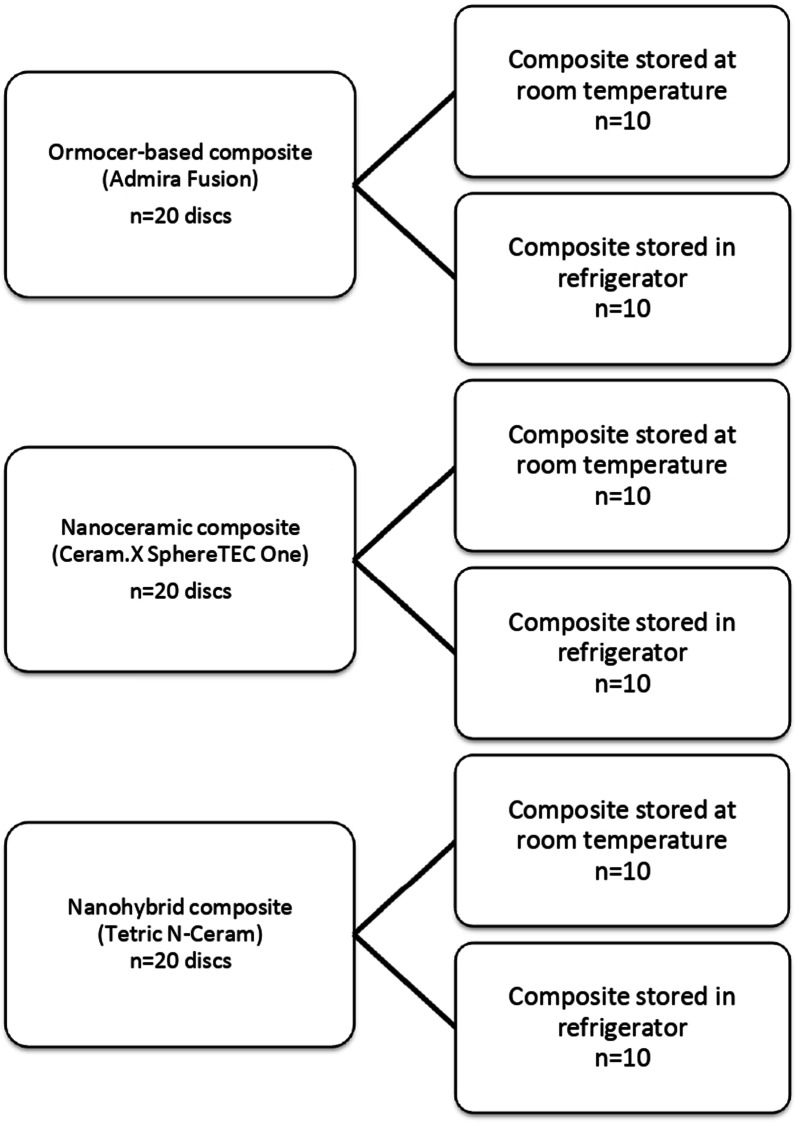
A diagram showing the grouping system of the different composite restorative materials for degree of conversion and microhardness tests

For DC test, 60 disc-shaped composite specimens were randomly divided into 3 main groups (n = 20) according to the restorative material used; an Ormocer-based composite, a nanoceramic composite, and a nanohybrid composite. Each group was divided into 2 subgroups (n = 10) according to the composite storage temperature; stored at room temperature or stored in the refrigerator at 4°–5 °C. The same study design was applied for microhardness test. The grouping system is illustrated in Fig. 1.

### Specimens preparation

**Fig. 2:**
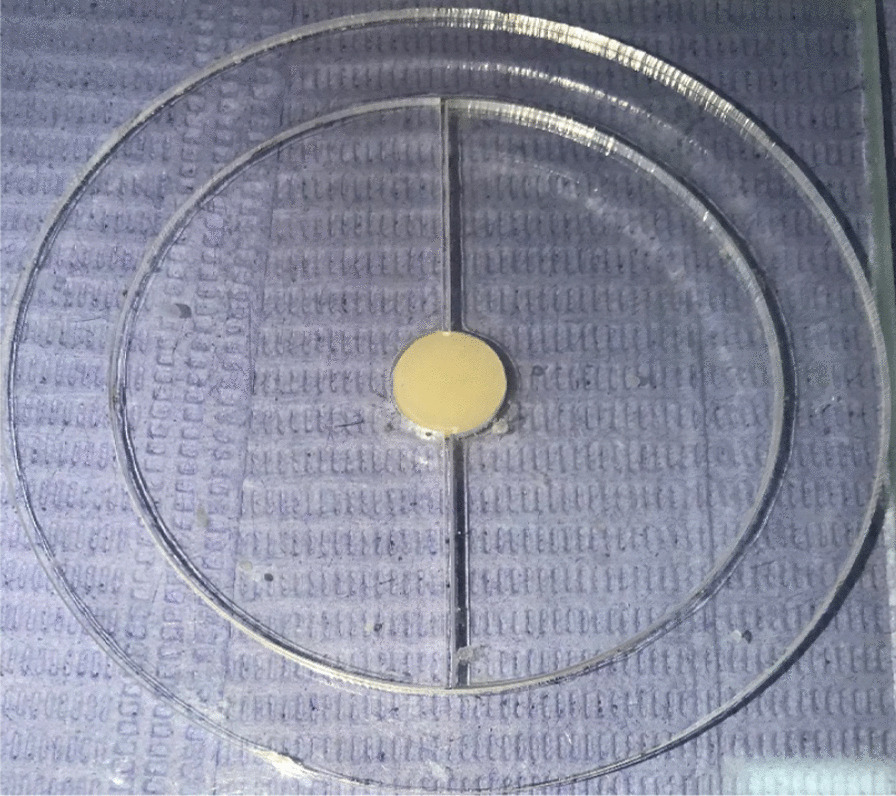
A cylindrical plastic mold (10 mm diameter × 2 mm thickness) used for fabricating the disc-shaped composite specimen

Specimens were prepared from each material using a specially designed cylindrical plastic mold (10 mm diameter × 2 mm thickness). Beneath the mold, a glass slide was held and covered with a transparent Mylar strip matrix. The composite restorative material was then packed into the mold using modeling instrument OptraSculpt (Ivoclar Vivadent AG, Schaan, Liechtenstein) till the mold space was filled completely. Another transparent Mylar strip matrix was applied on the surface and topped with another glass slide with pressure to achieve a flat surface [30]. Specimens were light-cured for 20 s through Mylar strip and glass slide using light emitting diode (LED) curing device (Elipar S10, 3M ESPE, St. Paul, MN, USA) with a wavelength between 430 and 480 nm and a light intensity of 1200 mW/cm^2^ as measured by the built-in light meter. The tip of the light-curing device was placed perpendicularly to the specimen’s surface with direct contact with the glass slide. To ensure uniform curing, 1 mm distance between the composite material and the light-curing device was standardized [31]. After glass slide removal, additional curing for both sides of the specimen was performed for 20 s. After every five specimens’ preparations, the light output was checked using built-in light meter (Fig. 2).

Regarding refrigerated composite materials, composite syringes were stored in the refrigerator for at least 30 min in order to stabilize the temperature of 4–5 °C. A composite syringe was then removed from the refrigerator and the composite material was immediately applied to the mold before its temperature changed appreciably. The composite syringe was returned to the refrigerator and replaced by another refrigerated syringe to make another specimen.

Finishing of the specimens was performed using high-speed diamond finishing instruments (4092.314, Komet, Brasseler, Lemgo, Germany) under copious air–water cooling. To achieve a smooth surface, flexible discs (Sof-Lex XT Pop On, 3M ESPE, St. Paul, MN, USA) and aluminum oxide impregnated cups (Enhance, Dentsply Caulk, Milford DE, USA) were used [32, 33]. Specimens were kept in a dry dark condition at 37 °C for 24 h prior to testing.

### Testing

#### Degree of conversion test

A Fourier-transform infrared spectrometer (FTIR) coupled to an attenuated total reflectance (ATR) crystal (Nicolet iS10 FTIR Spectrometer, Thermo Scientific, Madison, WL, USA) was used to measure the DC [34]. Composite specimens were held against the ATR crystal. Accordingly, an uncured specimen for each composite restorative material at both storage temperatures was also measured. All obtained spectra of both uncured and cured composite materials were recorded in absorbance mode in the 4000–400 cm^−1^ wave number range, while the spectral resolution was 4 cm^−1^. The absorbance intensities (peak heights) of the aliphatic double carbon–carbon bonds (C=C) stretching vibrations at 1637 cm^−1^, and aromatic single carbon–carbon bonds (C–C) stretching vibrations (internal standard) at 1608 cm^−1^ for nanohybrid and nanoceramic composites or 1588 ± 4 cm^−1^ for Ormocer-based composite were determined by a baseline method. Calculation of DC percentage of each tested specimen was performed according to the following equation; DC% = 1 − Cured (1637 cm^−1^/internal standard)/uncured (1637 cm^−1^/internal standard) × 100 [35].

#### Microhardness test

**Fig. 3:**
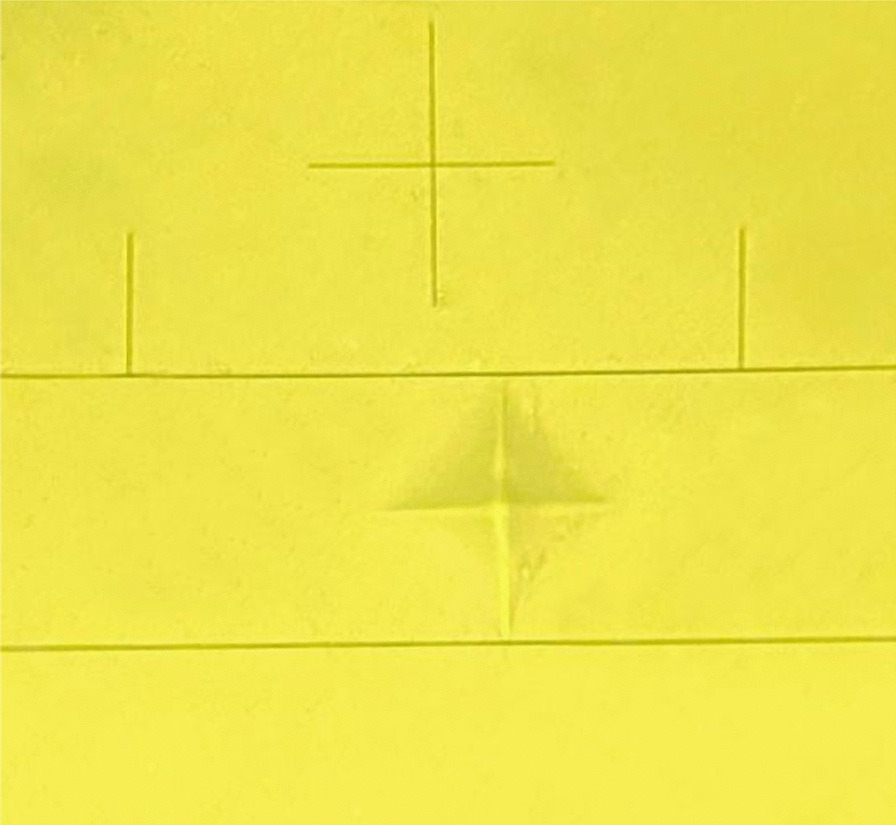
A photograph showing pyramidal-shape indentation performed by micro indenter of Micro-Vickers hardness tester

Vickers microhardness was evaluated at room temperature by micro-Vickers hardness tester (JINAN PRECISION TESTING EQUIPMENT CO., Model HV-1000 LTD, China). Five indentations were performed under a load of 50 gf with a dwell time of 10 s using a square base diamond pyramid-shaped micro-indenter with a 136° angle between its faces (Fig. 3). The indentations were performed at equal spaces on the surface of each specimen, each with a distance no closer than 1 mm to the adjacent indentations or the specimen margin. The five hardness measurements for each specimen were then averaged and reported as a single value. Vickers hardness number was automatically calculated using the following equation; VHN = 1.8544P/d^2^ where VHN is the Vickers hardness number in Kgf/mm^2^, P is the applied load in Kgf and d is the average length of the indentations’ diagonals in mm [36, 37].

### Statistical analysis

Data were tabulated and coded using (Microsoft Excel, 2016). The extracted data were analyzed using the Statistical Package for the Social Sciences (IBM-SPSS, version 24, Armonk, NY, USA). The distribution of data was statistically checked by Shapiro–Wilk test. A parametric Analysis of variance (ANOVA) was used for comparing more than two groups followed by post-hoc LSD test for in-between groups’ comparisons, and independent t-test for comparing two quantitative data. The level of significance was set at *p* < 0.05.

## Results

Shapiro–Wilk test revealed that all data showed no extreme deviation from normal distribution. Therefore, a parametric analysis of variance test (One-Way ANOVA) was conducted.

### Degree of conversion test

The outcome of One-Way ANOVA test revealed that there was a statistically significant difference among the studied groups at both storage temperatures (*p* < 0.05) with the highest mean values for Admira Fusion group followed by Ceram.X SphereTEC and Tetric N Ceram groups. The results of post-hoc LSD test showed that there was a statistically significant difference at both storage temperatures between the means of Admira Fusion and Tetric N Ceram groups (*p* < 0.05). Moreover, a statistically significant difference was noted between the means of Ceram.X SphereTEC One and Tetric N Ceram groups (*p* < 0.05). However, no statistically significant difference was found between the means of Admira Fusion and Ceram.X SphereTEC One groups (*p* > 0.05) as illustrated in Table 2. The results of Independent-t test revealed that there were statistically significant differences between all room-stored groups and their counterparts of refrigerator-stored groups for all composite restorative materials (*p* < 0.05).

**Table 2:** Means (N) and standard deviation (SD) values of DC after room temperature storage and refrigeration for different groups (each, n = 10)

Groups	Degree of conversion (room temperature)		Degree of conversion (refrigerator)	
	Mean ± SD (%)	Statistical post hoc category	Mean ± SD (%)	Statistical post hoc category
Admira F	64.12 ± 7.34	A	52.11 ± 5.78	A
Ceram X	59.22 ± 6.44	A	48.08 ± 5.01	A
Tetric N	52.35 ± 5.82	B	37.41 ± 4.13	B

Different letters indicate significant difference at level of significance test *p* < 0.05

### Microhardness test

One-Way ANOVA test showed that there was a statistically significant difference among the studied groups at both storage temperatures (*p* < 0.05) with the highest mean values for Admira Fusion group followed by Ceram.X SphereTEC and Tetric N Ceram groups. The results of post-hoc LSD test revelaed that there was a statistically significant difference at both storage temperatures between the means of Admira Fusion and Tetric N Ceram groups (*p* < 0.05). A statistically significant difference was found also between the means of Ceram.X SphereTEC One and Tetric N Ceram groups (*p* < 0.05). Conversely, no statistically significant difference was noted between the means of Admira Fusion and Ceram.X SphereTEC One groups (*p* > 0.05) as illustrated in Table 3. The results of Independent-*t* test showed that there were statistically significant differences between all room-stored groups and their counterparts of refrigerator-stored groups for all composite restorative materials (*p* < 0.05).

**Table 3:** Means (N) and standard deviation (SD) values of microhardness after room temperature storage and refrigeration for different groups (each, n = 10)

Groups	Micro hardness (room temperature)		Micro hardness (refrigerator)	
	Mean ± SD (kgf/mm^2^)	Statistical post hoc category	Mean ± SD (kgf/mm^2^)	Statistical post hoc category
Admira F	61.51 ± 6.08	A	54.67 ± 4.76	A
Ceram X	60.19 ± 5.26	A	53.14 ± 4.33	A
Tetric N	45.19 ± 4.35	B	35.99 ± 3.76	B

Different letters indicate significant difference at level of significance test *p* < 0.05

## Discussion

This study was conducted to focus on the impact of refrigeration on composite properties as the refrigerated storage of composite materials is a daily practice. Moreover, there is no sufficient information about the effect of storage temperature on Ormocer-based composites which might be attributed to the recent introduction of the material and lack of conducted studies evaluating this certain point. Therefore, an Ormocer-based and a nanoceramic composites were compared to a conventional nanohybrid based composite. To minimize the variability in the current study, the selected materials where all of approximate filler loading in order to exert more focus on the relation between the evaluated properties and the difference in matrix formulation.

In this in vitro study, the disc-shaped composite specimens were fabricated by specially designed cylindrical plastic mold to prevent stickiness of the composite materials during specimens’ preparations. The thickness of composite specimens was 2-mm to ensure appropriate light penetration and uniform polymerization [38]. Composite specimens were kept in a dry dark condition for 24 h after light curing before testing to prohibit any light passage to the made samples [39]. As well, to reach the maximum DC as considerable increase in DC takes place even after the removal of the light curing source, lasting up to 24 h after polymerization [40]. To control the parameters which may inadvertently affect the final results, all tested specimens were submitted to a standardized fabrication technique, light-curing intensity, light-exposure time, and curing distance between the specimen’s surface and the light curing device’s tip.

DC was evaluated directly using a FTIR-ATR method being a well-accepted and a widely used as it eliminates the need of sample grinding into powder or preparing potassium bromide (KBr) plates which simplifies sample preparation and allows curing under conditions closer to clinical cases. Moreover, this technique permits repeated analyses of the same location on a surface, thus ensuring high comparability between spectra before and after curing [41]. The area and amplitude ratios between the two main reference peaks (C=C absorption bands at 1607 cm^−1^ and 1637 cm^−1^) were used to determine the DC of resin composites. The only exception was applied to the Ormocer-based composite; Admira Fusion considering the peak position at 1588 ± 4 cm^−1^ as the reference peak [42].

Vickers microhardness test was used for this study as it is relatively simple, suitable for composite materials due to its higher stability, and reliable for obtaining adequate results [39]. Vickers microhardness analysis is reported as an indicator for the degree of polymerization of resin composites and used commonly as indirect technique to evaluate DC. Direct evaluation of composites’ DC, when combined with measuring the microhardness as a way to indirectly evaluate the DC has proven to be effective in characterizing the behavior of composite materials in dental practice [43]. The outcome of this study revealed that the microhardness results were generally correlated with the results of DC, suggesting that the highly polymerized composites with efficient polymeric network and high cross-linking have been shown to exhibit better microhardness. This comes in agreement with previous studies [44, 45] which showed that there was a correlation between hardness and DC independently of filler type and content. The statistically significant differences in DC and microhardness tests among all tested composite materials at both storage temperatures might be attributed to the difference in the matrix formulation chemistry of these materials which is considered an influential parameter in determining the final outcomes [46].

Both Ormocer-based and nanocermaic composites share the same Ormocer molecule in their matrix composition leading to a significant higher DC when compared to the nanohybrid composite. Ormocer molecule allows for formation of an inorganic link of siloxane (Si–O–Si) network through hydrolysis and polycondensation reactions. This results in a long inorganic ceramic polysiloxane matrix with lateral organic units, which can react during conventional light-activated polymerization [47]. The numerous organic polymerizable units for Ormocers might increase the probability of interactions and chemical bonding with neighbor molecules and thus increased the degree of cross-linking and monomer conversion. Whilst, the Bis-GMA molecule in nanohybrid composite has only two polymerizable units [48]. Conversely, Andrzejewska [49] reported a reduced double bond conversion for Ormocers. Their explanation was based on the denser network of Ormocer in which the double bonds are less reachable upon polymerization as a result of the steric hindrance of the Ormocer matrix as well as the glass filler particles.

The significant lower DC results for all refrigerator-stored groups when compared to their corresponding room-stored groups may be attributed to the effect of the low temperature in increasing the material’s viscosity. This may have decreased the movement of monomers and retarded the speed of the polymerization reaction. Consequently, a lower extent of the monomer conversion has been achieved [50]. Daronch et al. [51] reported that refrigerator-stored composites should not be used clinically until reaching room temperature at least.

Regarding microhardness, the filler particles with natural properties for Ormocer-based composite and nanoceramic composite could have increased the microhardness values via intense ionic inter-atomic bonds, which might explain such insignificantly different results [52]. However, the significant higher values for these both composites when compared to the nanohybrid composite might be attributed to the reported higher DC in addition to the accompaniment of the high-density organic matrix with hard glass fillers yielding a structure approximately as hard as glass. The spherical morphology of nanoceramic composite’s fillers could be the reason for the increased packing of particles which may have increased the hardness [53]. The significant decrease in the microhardness values of refrigerator-stored groups could be probably linked to the effect of low temperature which impaired the polymerization quality reflecting low microhardness values. This coincides with Osternack et al. [54] who reported that refrigerated tested composite materials showed statistically lower hardness values in comparison to their counterparts that polymerized in room temperature. On the contrary, this outcome disagreed with Torres et al. [55] who reported that composite cooling at 5 °C did not significantly affect the microhardness results.

## Conclusions

Refrigeration of resin-composite might have a deleterious effect on DC and microhardness of the tested composite restorative materials with different matrix systems. Moreover, the differences in the formulations of composite matrix have a potential impact on DC and microhardness. Ormocer-based and nanocermaic composites exhibited higher DC and microhardness values when compared to nanohybrid composite. A direct correlation exists between DC and microhardness regarding differences in restorative materials as well as storage temperature. Further investigations are still needed to fully assess the effect of refrigeration on polymerization shrinkage, fracture resistance, and wear rates of composite restorative materials to achieve the appropriate clinical performance. As no exact declaration regarding the right time to use composite restorative materials after removing it from the refrigerator, a comparison between immediate and delayed refrigeration has to be performed.

## Data Availability

The datasets used and/or analysed during the current study are available from the corresponding author on reasonable request.

## Abbreviations

DC: Degree of conversion
Ormocer: Organically modified ceramic
Bis-GMA: Bisphenol A-glycidyl methacrylate
LED: Light-emitting diode
FTIR: Fourier-transform infrared spectrophotometer
ATR: Attenuated total reflectance
C=C: Double carbon–carbon bonds
C–C: Single carbon–carbon bonds
VHN: Vickers hardness number
KBr: Potassium bromide
Bis-EMA: Ethoxylated bisphenol-A dimethacrylate
TEGDMA: Triethylene glycol dimethacrylate
UDMA: Urethane dimethacrylate
Admira F: Admira Fusion
Ceram X: Ceram.X SphereTEC One
Tetric N: Tetric N Ceram

## References

[1] Moraschini V, Fai CK, Alto RM, Dos Santos GO (2015). Amalgam and resin composite longevity of posterior restorations: a systematic review and meta-analysis. J Dent.

[2] Yadav R, Kumar M (2020). Investigation of the physical, mechanical and thermal properties of nano and microsized particulate-filled dental composite material. J Compos Mater.

[3] Yadav R, Kumar M (2019). Dental restorative composite materials: a review. J Oral Biosci.

[4] Yadav R (2021). Analytic hierarchy process-technique for order preference by similarity to ideal solution: a multi criteria decision-making technique to select the best dental restorative composite materials. Polym Compos.

[5] Yadav R (2022). Fabrication, characterization, and optimization selection of ceramic particulate reinforced dental restorative composite materials. Polym Polym Compos.

[6] Meena A, Bisht D, Yadav R, Saini S, Dangayach GS, Patnaik A, Meena ML (2022). Fabrication and characterization of micro alumina zirconia particulate filled dental restorative composite materials. Polym Compos.

[7] Yadav R, Lee HH (2022). Ranking and selection of dental restorative composite materials using FAHP-FTOPSIS technique: an application of multi criteria decision making technique. J Mech Behav Biomed Mater.

[8] Yadav R, Meena A (2022). Mechanical and two-body wear characterization of micro-nano ceramic particulate reinforced dental restorative composite materials. Polym Compos.

[9] Yadav R, Meena A (2022). Comparative study of thermo-mechanical and thermo gravimetric characterization of hybrid dental restorative composite materials. Proc Inst Mech Eng L: J Mater Des Appl.

[10] Antonucci JM, Dickens SH, Fowler BO, Xu HH, McDonough WG (2005). Chemistry of silanes: interfaces in dental polymers and composites. J Res Natl Inst Stand Technol.

[11] Cramer N, Stansbury J, Bowman CN (2011). Recent advances and developments in composite dental restorative materials. J Dent Res.

[12] Milosevic M (2016). Polymerization mechanics of dental composites–advantages and disadvantages. Procedia Eng.

[13] Karabela MM, Sideridou ID (2008). Effect of the structure of silane coupling agent on sorption characteristics of solvents by dental resin-nanocomposites. Dent Mater.

[14] Yoshinaga K, Yoshihara K, Yoshida Y (2021). Development of new diacrylate monomers as substitutes for Bis-GMA and UDMA. Dent Mater.

[15] Randolph LD, Palin WM, Leloup G, Leprince JG (2016). Filler characteristics of modern dental resin composites and their influence on physico-mechanical properties. Dent Mater.

[16] Cho E, Sadr A, Inai N, Tagami J (2011). Evaluation of resin composite polymerization by three dimensional micro-CT imaging and nanoindentation. Dent Mater.

[17] Goracci C, Cadenaro M, Fontanive L, Giangrosso G, Juloski J, Vichi A, Ferrari M (2014). Polymerization efficiency and flexural strength of low-stress restorative composites. Dent Mater.

[18] Mahmoud SH, Ali AK, Hegazi HA (2014). A three-year prospective randomized study of silorane- and methacrylate-based composite restorative systems in class II restorations. J Adhes Dent.

[19] Marghalani HY, Watts DC (2013). Viscoelastic stability of resin-composites aged in food-simulating solvents. Dent Mater.

[20] Moszner N, Gianasmidis A, Klapdohr S, Fischer UK, Rheinberger V (2008). Sol–gel materials: 2. Light-curing dental composites based on ormocers of cross-linking alkoxysilane methacrylates and further nano-components. Dent Mater.

[21] Bacchi A, Feitosa VP, da Silva Fonseca ASQ, Cavalcante LMA, Silikas N, Schneider LFJ (2015). Shrinkage, stress, and modulus of dimethacrylate, ormocer, and silorane composites. J Conserv Dent.

[22] Rodriguez A, Yaman P, Dennison J, Garcia D (2017). Effect of light-curing exposure time, shade, and thickness on the depth of cure of bulk fill composites. Oper Dent.

[23] Jafarzadeh-Kashi TS, Mirzaii M, Erfan M, Fazel A, Eskandarion S, Rakhshan V (2011). Polymerization behavior and thermal characteristics of two new composites at five temperatures: refrigeration to preheating. J Adv Prosthodont.

[24] Eshmawi YT, Al-Zain AO, Eckert GJ, Platt JA (2018). Variation in composite degree of conversion and microflexural strength for different curing lights and surface locations. J Am Dent Assoc.

[25] Al Sunbul H, Silikas N, Watts DC (2016). Surface and bulk properties of dental resin-composites after solvent storage. Dent Mater.

[26] Lucey S, Lynch CD, Ray NJ, Burke FM, Hannigan A (2010). Effect of pre-heating on the viscosity and microhardness of a resin composite. J Oral Rehabil.

[27] Castro FLAD, Campos BB, Bruno KF, Reges RV (2013). Temperature and curing time affect composite sorption and solubility. J Appl Oral Sci.

[28] Sabbagh J, Nabbout F, Jabbour E, Leloup G (2018). The effect of expiration date on mechanical properties of resin composites. J Int Soc Prev Community Dent.

[29] Borges GA, Spohr AM, Oliveira WJD, Correr-Sobrinho L, Correr AB, Borges LH (2006). Effect of refrigeration on bond strength of self-etching adhesive systems. Braz Dent J..

[30] Al-Qahatani YM, Al-Omari M, Mathew ST, Al-Qarni MAJT (2020). Degree of conversion of nanoceramic and microhybrid composites activated by different polymerization modes at different intervals: an in vitro comparative study. J Contemp Dent Pract.

[31] Moyin S, Lahiri B, Sam G, Nagdev P, Kumar NN (2020). Evaluation of the impact of acidic drink on the microhardness of different esthetic restorative materials: an in vitro study. J Contemp Dent Pract.

[32] Mahmoud S, El-Embaby A, AbdAllah A (2014). Clinical performance of ormocer, nanofilled, and nanoceramic resin composites in Class I and Class II restorations: a three-year evaluation. Oper Dent.

[33] Cetin A, Unlu N, Cobanoglu N (2013). A five-year clinical evaluation of direct nanofilled and indirect composite resin restorations in posterior teeth. Oper Dent.

[34] Collares FM, Portella FF, Leitune VCB, Samuel SMW (2014). Discrepancies in degree of conversion measurements by FTIR. Braz Oral Res.

[35] Tauböck TT, Jäger F, Attin T (2019). Polymerization shrinkage and shrinkage force kinetics of high-and low-viscosity dimethacrylate-and ormocer-based bulk-fill resin composites. Odontology.

[36] Comba A, Scotti N, Maravić T, Mazzoni A, Carossa M, Breschi L, Cadenaro M (2020). Vickers hardness and shrinkage stress evaluation of low and high viscosity bulk-fill resin composite. Polymers (Basel).

[37] Poggio C, Viola M, Mirando M, Chiesa M, Beltrami R, Colombo M (2018). Microhardness of different esthetic restorative materials: evaluation and comparison after exposure to acidic drink. Dent Res J.

[38] Hubbezoglu I, Bolayir G, Dogan OM, Dogan A, Özer A, Bek B (2007). Microhardness evaluation of resin composites polymerized by three different light sources. Dent Mater J.

[39] Dias M-F, Espíndola-Castro L-F, Lins-Filho P-C, Teixeira H-M, Silva C-H-V, Guimarães R-PJ (2020). Influence of different thermopolymerization methods on composite resin microhardness. J Clin Exp Dent.

[40] Harp YS, Montaser MA, Zaghloul NM (2022). Flowable fiber-reinforced versus flowable bulk-fill resin composites: degree of conversion and microtensile bond strength to dentin in high C-factor cavities. J Esthet Restor Dent.

[41] Kim I-H, Son JS, Min BK, Kim YK, Kim K-H, Kwon T-Y (2016). A simple, sensitive and non-destructive technique for characterizing bovine dental enamel erosion: attenuated total reflection Fourier transform infrared spectroscopy. Int J Oral Sci.

[42] Bolaños-Carmona V, Benavides-Reyes C, González-López S, González-Rodríguez P, Alvarez-Lloret P (2020). Influence of spectroscopic techniques on the estimation of the degree of conversion of bulk-fill composites. Oper Dent.

[43] Oberholzer TG, Grobler SR, Pameijer CH, Hudson AP (2003). The effects of light intensity and method of exposure on the hardness of four light-cured dental restorative materials. Int Dent J.

[44] Bouschlicher MR, Rueggeberg FA, Wilson BM (2004). Correlation of bottom-to-top surface microhardness and conversion ratios for a variety of resin composite compositions. Oper Dent.

[45] Galvão MR, Caldas SGFR, Bagnato VS, de Souza Rastelli AN, de Andrade MF (2013). Evaluation of degree of conversion and hardness of dental composites photo-activated with different light guide tips. Eur J Dent.

[46] da Silva EM, Miragaya L, Noronha-Filho JD, Amaral CM, Poskus LT, Guimarães JGA (2016). Characterization of an experimental resin composite organic matrix based on a tri-functional methacrylate monomer. Dent Mater.

[47] Monsarrat P, Garnier S, Vergnes J-N, Nasr K, Grosgogeat B, Joniot S (2017). Survival of directly placed ormocer-based restorative materials: a systematic review and meta-analysis of clinical trials. Dent Mater.

[48] Torres CR, Augusto MG, Mathias-Santamaria IF, Di Nicoló R, Borges AB (2020). Pure ormocer vs methacrylate composites on posterior teeth: a double-blinded randomized clinical trial. Oper Dent.

[49] Andrzejewska E (2001). Photopolymerization kinetics of multifunctional monomers. Prog Polym Sci.

[50] Calheiros FC, Daronch M, Rueggeberg FA, Braga RR (2014). Effect of temperature on composite polymerization stress and degree of conversion. Dent Mater.

[51] Daronch M, Rueggeberg F, De Goes M (2005). Monomer conversion of pre-heated composite. J Dent Res.

[52] Bayraktar ET, Atali PY, Korkut B, Kesimli EG, Tarcin B, Turkmen C (2021). Effect of modeling resins on microhardness of resin composites. Eur J Dent.

[53] Blackham JT, Vandewalle KS, Lien W (2009). Properties of hybrid resin composite systems containing prepolymerized filler particles. Oper Dent.

[54] Osternack FH, Caldas DB, Rached RN, Vieira S, Platt JA, Almeida JBD (2009). Impact of refrigeration on the surface hardness of hybrid and microfilled composite resins. Braz Dent J.

[55] Torres CR, Caneppele T, Borges AB, Torres A, Araújo MA (2011). Influence of pre-cure temperature on Vickers microhardness of resin composite. Int J Contemp Dent.

